# Correction: Musumeci et al. Intranasal Administration of a TRAIL Neutralizing Monoclonal Antibody Adsorbed in PLGA Nanoparticles and NLC Nanosystems: An In Vivo Study on a Mouse Model of Alzheimer’s Disease. *Biomedicines* 2022, *10*, 985

**DOI:** 10.3390/biomedicines12112447

**Published:** 2024-10-25

**Authors:** Teresa Musumeci, Giulia Di Benedetto, Claudia Carbone, Angela Bonaccorso, Giovanni Amato, Maria Josè Lo Faro, Chiara Burgaletto, Giovanni Puglisi, Renato Bernardini, Giuseppina Cantarella

**Affiliations:** 1 Laboratory of Drug Delivery Technology, Department of Drug and Health Sciences, University of Catania, 95125 Catania, Italy; tmusumec@unict.it (T.M.); ccarbone@unict.it (C.C.); abonaccorso@unict.it (A.B.); gamato@unict.it (G.A.); gpuglisi@unict.it (G.P.); 2 Department of Biomedical and Biotechnological Sciences, Section of Pharmacology, University of Catania, 95123 Catania, Italy; giulia.dibenedetto@unict.it (G.D.B.); chiaraburg@hotmail.it (C.B.); gcantare@unict.it (G.C.); 3 Dipartimento di Fisica e Astronomia “Ettore Majorana”, Università di Catania, Via Santa Sofia 64, 95123 Catania, Italy; mariajose.lofaro@unict.it; 4 CNR-IMM UoS Catania, Istituto per La Microelettronica e Microsistemi, Via Santa Sofia 64, 95123 Catania, Italy

## Error in Figure

Figure 5 in the original publication [1] was selected by mistake instead of the right one. The subfigures of panel A (a–c′; f′) and panel B (d–f′; g–i′) have now been replaced with the correct version, as the one originally submitted with the manuscript was uploaded by mistake. The corrected Figure 5 now appears below. The authors state that the scientific conclusions are unaffected. This correction was approved by the Academic Editor. The original publication has also been updated.

## Figures and Tables

**Figure 5:**
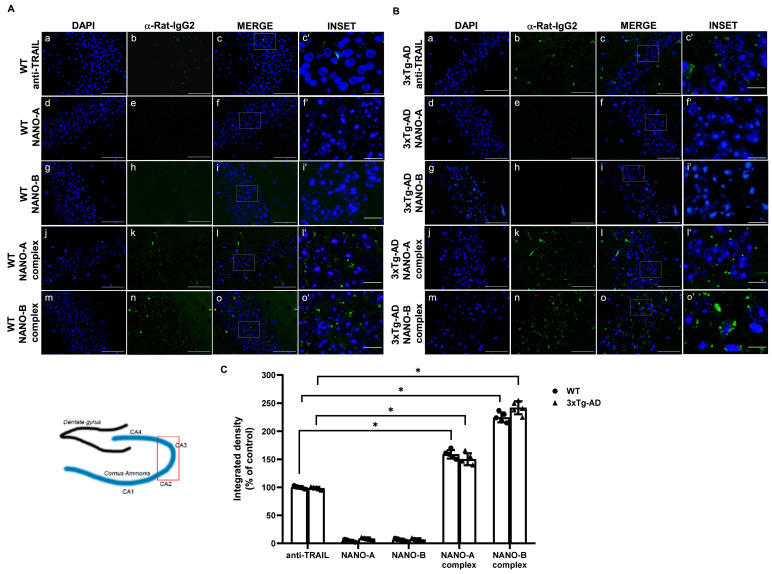
Comparison of polymeric or lipidic nanoparticles complexed with anti-TRAIL in the hippocampus of 3xTg-AD mice after intranasal administration. Representative immunofluorescence microscopy images showing the localization of the anti-TRAIL monoclonal antibody in the hippocampal sections from WT (**A**) or 3×Tg-AD (**B**) mice intranasally administered for 24 h with the anti-TRAIL monoclonal antibody, empty, or anti-TRAIL-loaded NANO-A, NANO-B nanoparticles. The fluorescence signal was detected by using a fluorescent secondary anti-rat IgG. The insets represent the respective areas magnified. (**C**) The densitometric count of fluorescence was performed with the aid of ImageJ software (available online: https://imagej.nih.gov/ij/ (accessed on 12 January 2022)) and represented as integrated density (% of control). Data are expressed as the mean ± SD. One-way ANOVA followed by Fisher’s LSD test was used for statistical analysis. * *p* < 0.05. (a–o scale bar = 50 µm; c’, f’, i’, l’, o’ scale bar = 10 µm).
